# A deep learning based multimodal fusion model for skin lesion diagnosis using smartphone collected clinical images and metadata

**DOI:** 10.3389/fsurg.2022.1029991

**Published:** 2022-10-04

**Authors:** Chubin Ou, Sitong Zhou, Ronghua Yang, Weili Jiang, Haoyang He, Wenjun Gan, Wentao Chen, Xinchi Qin, Wei Luo, Xiaobing Pi, Jiehua Li

**Affiliations:** ^1^ Clinical Research Institute, The First People’s Hospital of Foshan, Foshan, China; ^2^ R/D Center, Visionwise Medical Technology, Foshan, China; ^3^ Department of Dermatology, The First People’s Hospital of Foshan, Foshan, China; ^4^ Department of Burn and Plastic Surgery, Guangzhou First People's Hospital, South China University of Technology, Guangzhou, China; ^5^ Guangdong Medical University, Zhanjiang, China

**Keywords:** deep learning - artificial neural network, multimodal fusion, metadata, skin cancer, attention

## Abstract

**Introduction:**

Skin cancer is one of the most common types of cancer. An accessible tool to the public can help screening for malign lesion. We aimed to develop a deep learning model to classify skin lesion using clinical images and meta information collected from smartphones.

**Methods:**

A deep neural network was developed with two encoders for extracting information from image data and metadata. A multimodal fusion module with intra-modality self-attention and inter-modality cross-attention was proposed to effectively combine image features and meta features. The model was trained on tested on a public dataset and compared with other state-of-the-art methods using five-fold cross-validation.

**Results:**

Including metadata is shown to significantly improve a model's performance. Our model outperformed other metadata fusion methods in terms of accuracy, balanced accuracy and area under the receiver-operating characteristic curve, with an averaged value of 0.768±0.022, 0.775±0.022 and 0.947±0.007.

**Conclusion:**

A deep learning model using smartphone collected images and metadata for skin lesion diagnosis was successfully developed. The proposed model showed promising performance and could be a potential tool for skin cancer screening.

## Introduction

Skin cancer is one of the most common types of cancer. The number of skin cancer diagnosed all over the world reached 1.2 million in 2020 (1). Skin cancer is caused by damaged skin cells or abnormal growth of skin cells, which is closely related to excessive exposure to ultraviolet radiation, chemical carcinogens, and radioactive radiation (2). Early detection and treatment can improve the survival of patients. Dermoscope is a powerful tool used to observe pigmented skin disorders (2). By examining dermoscopic images, dermatologists can diagnose and grade a patient's lesion condition. However, reading dermoscopic images mainly relies on the experience and subjective judgments of dermatologists, which may introduce errors in the diagnosis process. Moreover, the cost of dermoscopy is relatively high, which imposes a considerable burden on patients in some underdeveloped country. Therefore, it is necessary to develop an automated tool to help to diagnose skin cancer and disease using affordable devices such as smartphone. Such a tool can reduce the cost, shorten the waiting time, and improve the accuracy of diagnosis.

Deep learning has emerged as a promising technology of artificial intelligence in recent years. With the continuous progress in the field of deep learning, artificial intelligence has made great breakthroughs in the field of medicine. Researchers have also shown positive outcomes in the smart diagnosis of skin cancer based on medical images. Various methods are proposed to diagnose skin cancers based on images (3). A mole classification system for early diagnosis of melanoma skin was proposed (4). The features were extracted according to the ABCD (5) rules of the lesions and classified them into common moles, rare moles and melanoma moles using a back-propagation feed-forward neural network. Aswin et al. (6) proposed a method for skin cancer detection based on Genetic Algorithm (GA) and Neural Network Algorithm, which divided lesion images into two categories: cancerous and non-cancerous. A skin cancer detection system based on convolutional neural network (CNN) was proposed by Mahbod et al. (7) who extracted deep features from pretrained deep CNNs for classification of skin diseases. Kalouche (8) proposed to finetune a pretrained deep CNN architecture VGG-16 and achieved an accuracy of 78%. A method combining a self-organizing neural network and a radial basis function (RBF) neural network was proposed to diagnose three different types of skin cancers with an accuracy of 93%, a significant improvement over traditional classifiers (9). Bisla et al. (10) proposed a deep learning method for data cleaning and a GAN method for data augmentation, which achieved 86.01% classification accuracy. Ali et al. also proposed a skin damage data enhancement method based on self-attention progressive GAN (11).

In real-world clinical setting, dermatologists make medical decision by considering a variety of information, including different types of imaging result, laboratory result, demographic information and patient feedback on their own feeling. To better utilize information from multiple sources, multi-modal fusion classification is introduced into the detection and classification of skin cancer. Cai et al. (12) proposed a multimodal transformer to fuse multimodal information. Chen et al. (13) proposed a skin cancer Multimodal Data Fusion Diagnosis Network (MDFNet) framework based on a data fusion strategy to effectively fuse clinical skin images with patient clinical data. Yap et al. (15) propose a method that combines multiple imaging modalities with patient metadata to improve the performance of automated skin lesion diagnosis. Li et al. proposed the MetaNet, which uses a sequence of 1D convolution on metadata to extract coefficients to assist visual features extracted from images in the classification task (16). Pacheco et al. developed a metadata processing block (MetaBlock) to fuse metadata with image data, which outperformed MetaNet and conventional feature concatenation method (17).

However, previous studies mainly focus the combination of metadata and image feature without exploring the underlying relationship between the two modalities. We argued that metadata provides extra information which can guide the interpretation of image. Similarly, image features also contain unique information which can guide the understanding of metadata. The two data modalities can facilitate each other to better unveil features that are most relevant to disease classification. We therefore proposed an attention-guided multimodal fusion network to better integrate imaging data and metadata for skin lesion diagnosis.

## Methods

### Model development

In medical diagnosis, clinicians are usually faced with multiple sources of information. Such information usually includes medical images and metadata (clinical or demographic supporting information that are not in the form of images). Medical decision is made after aggregating various aspects of information. In deep learning, the simplest method to combine information from different sources is channel concatenation. However, since imaging data is usually in higher dimensional than metadata, simply concatenating or adding them may not be the optimal solution for information fusion. We therefore proposed our method called multimodal fusion network (MMF-Net) to better solve the problem of image and metadata fusion.

### Experiment description

In this study, we used the images and meta information provided in PAD-UPES-20 as our experimental data (17). Data were obtained from the Dermatological and Surgical Assistance Program at the Federal University of Espírito Santo (UFES), and all samples were representative of skin lesions in patients and consisted of images and meta data. The dataset includes 2,298 images collected from smartphones including 6 different types of skin lesions. Each image also contains up to 21 clinical characteristics, including but not limited to age, lesion location, Fitzpatrick skin type, and lesion diameter. All data can be accessed on the following website https://data.mendeley.com/datasets/zr7vgbcyr2/1.1. In the dataset, among the 2,298 cases, 730 of them are actinic keratosis (ACK), 845 are Basal Cell Carcinoma (BCC), 52 are malignant melanoma (MEL), 244 are Melanocytic Nevus of Skin (NEV), 192 are Squamous Cell Carcinoma (SCC) and 235 are Seborrheic Keratosis (SEK).

### Model construction

To classify skin lesion types, each sample is given a smartphone photo and accompanying meta information, shown in Table 1. A convolutional neural network such as ResNet-50 was used to extract features from the smartphone image, denoted as *x_img_*. Metadata is preprocessed as follow: numerical features were kept the same; Boolean features were converted to 0 and 1. Categorical features were one-hot encoded. For example, for the gender attribute, the one-hot encoding is [1,0] for male, [0,1] for female and [0,0] for missing information. A multi-layer perceptron was applied to extract features from meta information, denoted as *x_meta_*. Our goal is to estimate the probability *y* of a skin lesion belonging to a certain class *c* in one of the six categories: Basal Cell Carcinoma (BCC), Squamous Cell Carcinoma (SCC), Actinic Keratosis (ACK), Seborrheic Keratosis (SEK), Melanoma (MEL), and Nevus (NEV), given the input image and meta information.

**Table 1:** Description of attributes in meta information.

Meta variable	Description	*P* value
Smoking		<0.001
Drinking		0.476
Father country	Which country the patient's father is from	<0.001
Mother country	Which country the patient's mother is from	0.012
Age		<0.001
Gender		0.032
Cancer history	If the patient or someone in their family had history of any type of cancer in the past	0.821
Skin cancer history	If the patient or someone in their family had history of skin cancer in the past	0.067
Pesticide	If the patient use pesticide	<0.001
Sewage system	If the patient has sewage system access in their home	0.019
Piped water	If the patient has piped water access in their home	0.029
Fitspatrick skin type		<0.001
Region	Living region	0.598
Diameter 1	Horizontal diameter of lesion	<0.001
Diameter 2	Vertical diameter of lesion	<0.001
Itch	If the lesion itches	<0.001
Grew	If the lesion has grown recently	<0.001
Hurt	If the lesion hurts	<0.001
Changed	If the lesion has changed recently	<0.001
Bleed	If the lesion has bled	<0.001
Elevation	If the lesion has an elevation	<0.001


$$\hat{y}=p(y=c|x_{img},x_{meta})$$

**Figure 1:**
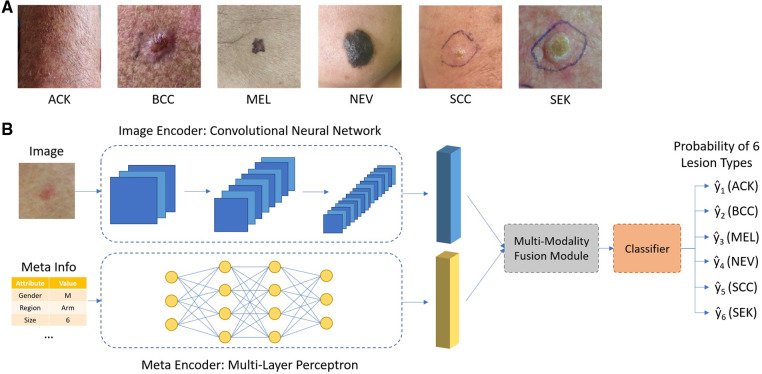
(**A**) Typical images of different types of skin lesions; (**B**) overall network architecture of the proposed network.

The overall design of the network is shown in Figure 1. The image encoder and meta encoder extract features from image and meta information, respectively. The extracted features are then fused together by the multimodal fusion module and passed into the classifier module composed of a fully connected layer. The final output is a six-channel vector representing the probability of six lesion types.

**Figure 2:**
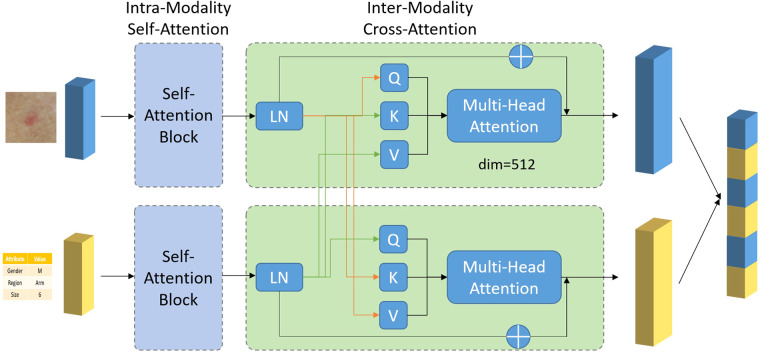
Network architecture of the proposed multimodal fusion module.

The simplest method of combining image and meta information, is by concatenating their corresponding features *x_img_* and *x_meta_* in the channel dimension or by summation. Yet these approaches essentially treat image and meta information as the same, ignoring the underlying differences between them. Moreover, there are inter-correlation between image and meta information, complementing of each other, which may be overlooked by simple combination. To exploit such intrinsic connection between two modalities, an attention-based multi-modality fusion module is proposed. Basically, what the attention mechanism does is to assign different weights to different features. Features with larger weights are considered to be more important in the diagnosis process and vice versa. One typical example for the use of attention mechanism is, if a specific body location is related to just one or few types of skin diseases, when such information is available as meta data, it could be used to directly suppress the prediction probability of other irrelevant diseases, thus guiding the network to focus on the rest possible types. The overall design of the fusion module, composed of intra-modality self-attention and inter-modality cross-attention, is shown in Figure 2.

### Intra-modality self-attention

There may exist irrelevant information in each modality (e.g., image background in image). To avoid irrelevant information to confuse the neural network and to better guide the network to focus on key features, a multi-head self-attention module is applied to the two features, respectively. A typical attention module is consisting of two steps, linearly projecting the input feature *x* into Query (Q), Key (K) and Value (V) vector, followed by multiplying *V* by the attention weight obtained from dot-product of *Q* and *K* to get the weighted feature *x’*, shown as below:

$$Q=W_{q}x,\;\;K=W_{k}x,\;\;V=W_{v}x$$

$$x^{\prime }=Softmax\left(\frac{QK^{T}}{\sqrt{d}}\right)V$$

After passing through the self-attention module, we obtained the weighted *x_img_* and *x_meta_*, which gives high weighting to cancer-relevant information and low weighting to irrelevant information.

### Inter-modality cross-attention

This module contains two paths. One path is designed to use meta information to guide the selection of most relevant information from image feature. The other path is designed the other way around, to use image feature to guide the selection of most relevant information from meta data. For the first path, a cross-attention module is designed with input vector Query and Value from *x_img_* projection and Key from *x_meta_*, shown as below:

$$Q_{1}=W_{q1}x_{img}^{\prime },\;\;K_{1}=W_{k1}x_{imeta}^{\prime },\;\;V_{1}=W_{v1}x_{img}^{\prime }$$

$$x_{img}^{\prime \prime }=Softmax\left(\frac{Q_{1}{K_{1}}^{T}}{\sqrt{d}}\right)V_{1}$$

For the second part, a cross-attention module is designed with input vector Query and Value from *x_meta_* projection and Key from *x_img_*, shown as below:

$$Q_{2}=W_{q2}x_{meta}^{\prime },\;\;K_{2}=W_{k2}x_{img}^{\prime },\;\;V_{2}=W_{v2}x_{meta}^{\prime }$$

$$x_{meta}^{\prime \prime }=Softmax\left(\frac{Q_{2}{K_{2}}^{T}}{\sqrt{d}}\right)V_{2}$$

We then concatenated *x_meta_’’* and *x_img_’’* to obtained the final feature vector *x_final_* that is passed into a fully connected layer and a softmax layer to output the probability of six lesion classes.

### Training and evaluation procedures

To ensure a fair comparison with other methods, we followed the experimental setup as that in Andre's work (14). We measured different methods’ performance by calculating the following metrics including, accuracy (ACC), balanced accuracy (BACC) and aggregated area under the curve (AUC). The balanced accuracy is calculated by the arithmetic mean of sensitivity and specificity. As we can see that the portion of different diseases in the dataset is imbalanced, balanced accuracy is therefore more favored in such case as accuracy may be biased. The aggregated area under the curve is obtained by computing the average AUC of all possible pairwise combinations of the six classes, which will not be biased by imbalanced class distribution in our data. Five-fold cross-validation stratified by the label's frequency was applied for each competing method, namely baseline feature concatenation, MetaNet, MetaBlock and our proposed method. In each split, the whole dataset is split into five folds. Four folds were used as training set and the remaining fold was used as test set. The process was repeated five times until each fold has been used as test set at least once. The averaged performance of the five folds were reported.

During training, we used the SGD optimizer with an initial learning rate of 0.001. The learning rate is reduced to 0.1 of its current value if training loss stop to decrease within 10 epochs. A total of 100 epochs were trained for each method. Data augmentations such as horizontal and vertical flipping, color jittering, gaussian noise and random contrast were applied on-the-fly during training. All codes were written in Python 3.6.10. PyTorch framework (v1.7.0) was used by constructed the neural network.

### Statistical analysis

Metadata attributes were examined by univariate analysis. Continuous variables were first examined by Shapiro–Wilk test to determine if they were normally distributed. Student *t* test (for normally distributed parameters) or Mann–Whitney *U* test (for non-normally distributed parameters) were used to identify statistically significant variable between different skin lesion types. Categorical variables were compared by chi-square test. To compare different methods’ performance, non-parametric Friedman test followed by the Wilcoxon test were used. *P* < 0.05 is consider to be statistically significant. The statistical analysis was performed with SPSS (IBM, version 26).

## Results

**Figure 3:**
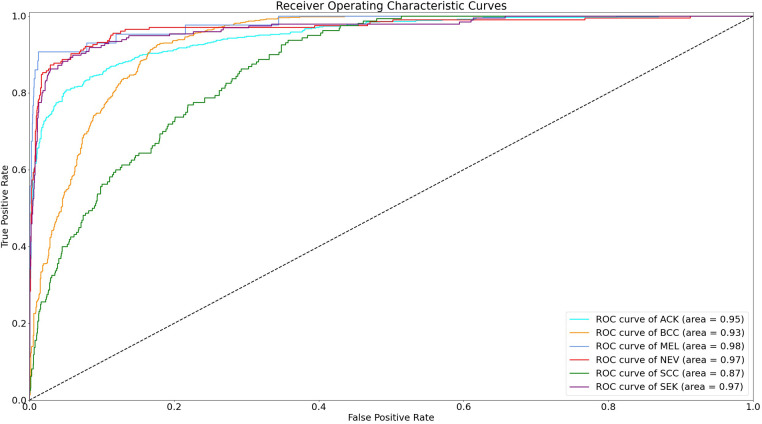
Receiver operating characteristic curves for different types of skin lesions.

**Figure 4:**
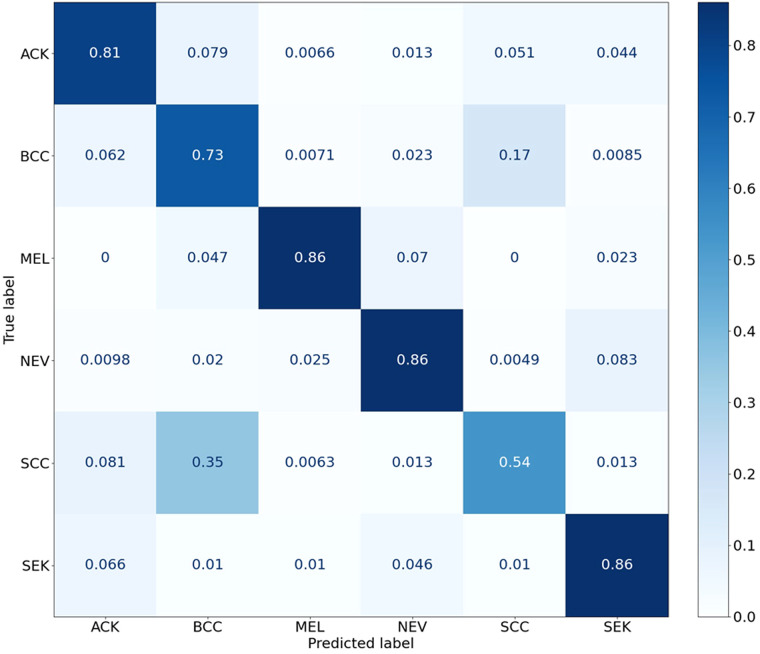
Confusion matrix of different types of skin lesions.

The statistical significance of the difference of metadata attributes between different lesion types is shown in Table 1. Most metadata attributes are significantly correlated with different types of skin lesion. The averaged five-fold cross-validation performance of each method is presented in Table 2. Our proposed method achieved the best performance in ACC (0.768 ± 0.022), BACC (0.775 ± 0.022) and AUC (s) metrics. We can observe that including meta information significantly improves the performance in terms of AUC (0.947 ± 0.007 vs. 0.901 ± 0.007, *P* < 0.001), ACC (0.768 ± 0.022 vs. 0.616 ± 0.051, *P* < 0.001) and BACC (0.775 ± 0.022 vs. 0.651 ± 0.050, *P* < 0.001), as shown in Table 3. Our proposed method utilized meta information in a more effective way, which significantly outperformed the other two methods MetaNet (*P* = 0.035) and MetaBlock (*P* = 0.028). The ROC curves for six lesion types are plotted in Figure 3. The proposed method achieved the best performance for MEL (AUC = 0.98), followed by NEV (AUC = 0.97), SEK (AUC = 0.97), ACK (AUC = 0.95) and BCC (AUC = 0.93). SCC is the most difficult one to identify, with AUC = 0.87. The averaged AUC of six disease types is 0.947. We further examined the failed cases. Figure 4 shows the averaged confusion matrix, from which we can see that SCC has the lowest accuracy because it is often mistaken as BCC. This is understandable as our images are collected from smartphone. Clinically, there is a tendency of SCC misdiagnosed as BCC. Using dermoscopy can improve the diagnostic accuracy (22). We further performed an ablation study to analyze different components’ effect on the final result. Table 4 shows the performance of the model without the inter-modality self-attention and model without the intra-modality cross-attention. This indicates that the two modules act synergistically to improve the fusion of image and metadata.

**Table 2:** Performance comparison of different methods.

	ACC	BACC	AUC
No metadata	0.616 ± 0.051	0.651 ± 0.050	0.901 ± 0.007
Concatenation	0.741 ± 0.014	0.728 ± 0.029	0.929 ± 0.006
MetaBlock	0.735 ± 0.013	0.765 ± 0.017	0.935 ± 0.004
MetaNet	0.732 ± 0.054	0.742 ± 0.019	0.936 ± 0.006
Our Method	0.768 ± 0.022	0.775 ± 0.022	0.947 ± 0.007

**Table 3:** Result of the Wilcoxon pair test for different methods.

Pair	*P* value
No metadata—our method	<0.001
MetaBlock—our method	0.028
MetaNet—our method	0.035

**Table 4:** Ablation study of the proposed method.

	ACC	BACC	AUC
w/o Self-attention	0.757 ± 0.026	0.765 ± 0.025	0.938 ± 0.008
w/o Cross-attention	0.743 ± 0.021	0.759 ± 0.021	0.936 ± 0.006
Full module	0.768 ± 0.022	0.775 ± 0.022	0.947 ± 0.007

## Discussion

We have developed a deep learning model to diagnose skin lesion by using clinical images and meta information obtained from smartphones. We proposed a new module which uses the combination of intra-modality self-attention and inter-modality cross-attention to better fuse image and metadata. The proposed model achieved promising performance on the public dataset, outperforming other state-of-the-art methods.

Compared with previous work (MetaNet, MetaFuse) on metadata fusion, our method has made two improvements. First, we used a multi-head self-attention module which calculates the feature correlation matrix to assign attention weights to different features within the same modality, removing irrelevant information in each modality. Second, unlike previous works where the attention is one-way (metadata feature ≥ image feature), our attention mechanism works two-way. Metadata features are used to guide the attention on image features and vice versa, image features are also used to guide the attention on metadata features. This has enabled our model to better exploit the information in each modality and outperform the other methods, as demonstrated in our experiments.

Many previous studies applying artificial intelligence to skin disease diagnosis focused on dermoscopy images only. However, dermoscope is not always accessible to many people in rural area or in underdeveloped country. In contrast, smartphone is a more accessible tool to many patients. Moreover, many patients tend to seek advices online instead of scheduling a visit to the clinic at the early stage of skin disease, which can delay their treatment and result in poor prognosis. The model developed in the current study can be integrated into a smartphone-based automatic screening app, which can help to identify high risk lesion and urge the patient for immediate treatment.

In real world clinical settings, dermatologists seldom make decision solely based on images. They usually make their judgement based on patient demographics (age, gender) and lesion characteristics (anatomical region) (18) which cannot be simply obtained from images. They also consider other risk factors such as cancer history, exposure to chemical and types of skin (19). Including meta information and multiple sources of data has been proved to be helpful for deep learning model in some previous studies (20, 21). In this study, we incorporated meta information into neural network and have showed that it can significantly improve the skin lesion diagnostic performance. Such information can be easily obtained in the form of online questionnaire which costs only a few clicks on the app from the user.

Though we have developed a promising model for skin lesion diagnosis, there are several limitations in our study. The number of included skin lesion types is relatively few compared to real world scenario. This can be improved by collecting data from more diseases in the future. External validation using data collected from different sites is needed before the model is put into clinical practice.

## Conclusion

We have developed a deep learning model to diagnose skin lesion using clinical images and meta information obtained from smartphones. A new module consisting of intra-modality self-attention and inter-modality cross-attention is proposed to better fuse image data and metadata and is shown to outperformed other state-of-the-art methods. Our proposed model could be integrated into smartphone as a potential and handy tool to screen for skin disease and skin cancer.

## Data Availability

The original contributions presented in the study are included in the article/Supplementary Material, further inquiries can be directed to the corresponding author/s.
